# Supplementary material for: Comparison study of image quality at various radiation doses for CT venography using advanced modeled iterative reconstruction
Source: PLoS One. 2021 Aug 31;16(8):e0256564. doi: 10.1371/journal.pone.0256564 (PMC8407572; doi:10.1371/journal.pone.0256564)
Supplement: Attachment — Submitted filename: Response to Reviewers.docx [file pone.0256564.s002.docx]

-------- Point-by-point response --------

Editor

Please re-address the comment 4 of Reviewer 2 about CarekV from last review. Since CarekV is a standard package on the scanner for the same purpose of this study, please add some discussion about the limitation of this study if the experiment suggested by the reviewer can't be conducted.

**Response:** Thank you for your comment. We have added the limitation as suggested “Fourth, we selected fixed 80 kVp and compared between specific radiation doses. Selection of automatic kVp change or fixed kVp is possible in Siemens CT. However, using the specific split of the tube dose in a dual source mode, we can only select a specific kVp (i.e, cannot use automatic kVp change).” (Editor).

Journal Requirements:

Please review your reference list to ensure that it is complete and correct. If you have cited papers that have been retracted, please include the rationale for doing so in the manuscript text, or remove these references and replace them with relevant current references. Any changes to the reference list should be mentioned in the rebuttal letter that accompanies your revised manuscript. If you need to cite a retracted article, indicate the article’s retracted status in the References list and also include a citation and full reference for the retraction notice.

**Response:** Thank you for your comment.

Reviewers' comments:

Reviewer's Responses to Questions

Comments to the Author

1. If the authors have adequately addressed your comments raised in a previous round of review and you feel that this manuscript is now acceptable for publication, you may indicate that here to bypass the “Comments to the Author” section, enter your conflict of interest statement in the “Confidential to Editor” section, and submit your "Accept" recommendation.

Reviewer #1: All comments have been addressed

Reviewer #2: (No Response)

2. Is the manuscript technically sound, and do the data support the conclusions?

The manuscript must describe a technically sound piece of scientific research with data that supports the conclusions. Experiments must have been conducted rigorously, with appropriate controls, replication, and sample sizes. The conclusions must be drawn appropriately based on the data presented.

Reviewer #1: Yes

Reviewer #2: Yes

3. Has the statistical analysis been performed appropriately and rigorously?

Reviewer #1: Yes

Reviewer #2: Yes

4. Have the authors made all data underlying the findings in their manuscript fully available?

The PLOS Data policy requires authors to make all data underlying the findings described in their manuscript fully available without restriction, with rare exception (please refer to the Data Availability Statement in the manuscript PDF file). The data should be provided as part of the manuscript or its supporting information, or deposited to a public repository. For example, in addition to summary statistics, the data points behind means, medians and variance measures should be available. If there are restrictions on publicly sharing data—e.g. participant privacy or use of data from a third party—those must be specified.

Reviewer #1: Yes

Reviewer #2: Yes

5. Is the manuscript presented in an intelligible fashion and written in standard English?

PLOS ONE does not copyedit accepted manuscripts, so the language in submitted articles must be clear, correct, and unambiguous. Any typographical or grammatical errors should be corrected at revision, so please note any specific errors here.

Reviewer #1: Yes

Reviewer #2: Yes

6. Review Comments to the Author

Please use the space provided to explain your answers to the questions above. You may also include additional comments for the author, including concerns about dual publication, research ethics, or publication ethics. (Please upload your review as an attachment if it exceeds 20,000 characters)

Reviewer #1: The authors have addressed my concerns. The major issue I had was that we cannot claim it was the advanced recon that allowed the dose reduction, I think the authors have changed the paper's verbiage to account for the fact their study never compared FBP low dose to ADMIRE low dose directly. Thanks.

Reviewer #2: (No Response)

7. PLOS authors have the option to publish the peer review history of their article (what does this mean?). If published, this will include your full peer review and any attached files.

If you choose “no”, your identity will remain anonymous but your review may still be made public.

Do you want your identity to be public for this peer review? For information about this choice, including consent withdrawal, please see our Privacy Policy.

Reviewer #1: Yes: Timothy P Szczykutowicz

Reviewer #2: No

**Response 1~7):** Thank you for your helpfu
